# Brain Metastasis After Curative Resection of Non-small Cell Lung Cancer: A Systematic Review and Meta-Analysis

**DOI:** 10.7759/cureus.101463

**Published:** 2026-01-13

**Authors:** Ryuichi Ohta, Kasumi Nishikawa, Kaoru Tanaka, Hidetoshi Hayashi

**Affiliations:** 1 Community Care, Unnan City Hospital, Unnan, JPN; 2 Family Medicine, Unnan City Hospital, Unnan, JPN; 3 Medical Oncology, Kindai University Faculty of Medicine, Sakai, JPN

**Keywords:** brain neoplasms, local neoplasm metastasis, magnetic resonance imaging, neoplasm recurrence, non-small cell lung carcinoma, risk factors

## Abstract

Metachronous brain metastasis is a clinically significant pattern of recurrence after curative-intent surgery for non-small cell lung cancer (NSCLC). The extent to which contemporary clinicopathological and molecular characteristics predict postoperative intracranial relapse, and whether routine postoperative neuroimaging might aid early detection, remains uncertain. We performed a systematic review and meta-analysis of studies reporting postoperative brain metastasis following curative resection for NSCLC. Fourteen retrospective cohort studies published between 2010 and 2025 were included, comprising 13,414 patients and 883 documented intracranial recurrence events. Data extraction focused on clinicopathological factors, molecular features, follow-up duration, and multivariable-adjusted hazard ratios (HRs). Only HRs derived from multivariable Cox regression models were pooled; studies reporting odds ratios without time-to-event adjustment were not combined quantitatively, ensuring comparability of effect estimates and minimizing confounding. The overall incidence of metachronous brain metastasis was approximately 6.6%. Routine postoperative brain imaging was implemented in only 28.6% of studies, whereas the majority relied on symptom-triggered neuroimaging. Adenocarcinoma was the predominant histology, accounting for 45-100% of cases. Although EGFR mutation was variably associated with increased intracranial recurrence, the pooled estimate demonstrated substantial heterogeneity (I² = 86.1%), limiting interpretability. In contrast, traditional clinicopathological factors-including nodal involvement, visceral pleural invasion, tumor differentiation, smoking history, and chronic pulmonary comorbidities-showed more consistent associations across multivariable models with low to moderate heterogeneity. Considerable heterogeneity in imaging strategies and molecular reporting was observed. Metachronous brain metastasis remains an important postoperative recurrence pattern in surgically treated NSCLC. Clinicopathological and host-related factors appear to be more consistently predictive than molecular features alone. Whether selected high-risk patients may benefit from scheduled rather than purely symptom-driven postoperative brain imaging warrants further prospective investigation. Standardized surveillance protocols and integration of clinical, pathological, and molecular determinants are essential to improve risk stratification and to facilitate earlier detection of intracranial relapse.

## Introduction and background

Non-small cell lung cancer (NSCLC) accounts for approximately 80-85% of all lung malignancies and remains the leading cause of cancer-related mortality worldwide despite advances in diagnostic and therapeutic strategies [1]. Surgical resection is the standard curative modality for patients with early-stage NSCLC, and improved radiological techniques - including low-dose computed tomography (LDCT) - have contributed to increasing detection rates of early-stage disease and prolonged survival [2,3]. Recent evidence from screening trials, including the SUMMIT study, has further emphasized the mortality benefit of early diagnosis through LDCT, underscoring the growing importance of postoperative management in surgically treated populations [3].

Although curative surgery offers favorable long-term survival in early-stage NSCLC, postoperative recurrence remains a clinically significant issue [4]. Among distant recurrence sites, the brain represents one of the most frequent and clinically devastating locations, often leading to neurological symptoms, impaired functional status, and poor quality of life [4]. Existing international and national guidelines, including the National Comprehensive Cancer Network (NCCN, version 2024-2025) and the American Society of Clinical Oncology (ASCO, most recent surveillance guidance), recommend routine postoperative surveillance with chest computed tomography, whereas scheduled brain imaging is not recommended for asymptomatic patients [4]. This recommendation is primarily based on the historically low detection yield of routine brain imaging in unselected postoperative populations, concerns regarding cost-effectiveness, and the lack of clear evidence demonstrating a survival benefit from routine neuroimaging in the absence of neurological symptoms. Consequently, postoperative brain metastases are frequently identified only after the onset of neurological manifestations, which may delay diagnosis and limit timely intervention in routine clinical practice.

In addition, the true incidence of postoperative metachronous brain metastasis remains uncertain due to variations in study design, surveillance strategies, and definitions of recurrence across published studies [5]. Moreover, advances in systemic therapy, including epidermal growth factor receptor (EGFR) tyrosine kinase inhibitors and immune checkpoint inhibitors, may influence recurrence patterns, including a potential impact on brain tropism [6,7]. While several studies have suggested associations between clinicopathological characteristics and brain recurrence, the available evidence remains heterogeneous and fragmented [8,9].

Importantly, a comprehensive synthesis focusing specifically on brain metastasis as the first site of recurrence after curative surgery is lacking. In contrast to prior reviews that pooled heterogeneous recurrence outcomes or unadjusted estimates, the present review was methodologically restricted to studies reporting multivariable-adjusted effect estimates, enabling a more rigorous evaluation of independent risk factors for postoperative intracranial relapse [10]. Consequently, risk stratification strategies capable of identifying high-risk individuals who may benefit from intensified surveillance remain insufficiently defined.

Furthermore, despite increasing availability of local and systemic treatment modalities - including stereotactic radiosurgery (SRS), whole-brain radiotherapy (WBRT), neurosurgical resection, and targeted systemic therapies - the optimal management of postoperative brain recurrence has not been clearly established, and high-quality evidence integrating treatment patterns and prognostic determinants remains limited [6,11].

Therefore, we conducted a systematic review and meta-analysis to determine the incidence of metachronous brain metastasis after curative resection for NSCLC, identify clinicopathological risk factors associated with postoperative brain recurrence, and summarize available evidence on treatment strategies and subsequent survival outcomes. Our findings aim to support future development of risk-adapted postoperative surveillance strategies, refine clinical decision-making, and establish an evidence-based foundation for further prospective investigation.

## Review

Study design

This study was conducted as a systematic review and meta-analysis following the Preferred Reporting Items for Systematic Reviews and Meta-Analyses (PRISMA) 2020 guidelines [12]. The protocol was prospectively registered in the International Prospective Register of Systematic Reviews (PROSPERO) (registry number: CRD420251248812). We included original research reporting postoperative brain metastasis among patients with curatively resected NSCLC.

Data sources and search strategy

A comprehensive literature search was conducted using PubMed, Embase, and Web of Science from January 2010 to October 2025. The search strategy incorporated Medical Subject Headings (MeSH) and free-text terms related to NSCLC, surgical resection, recurrence, and brain metastasis. The following search string was applied: (“lung cancer” OR “non small cell lung cancer” OR NSCLC) AND (“resection” OR “surgical resection” OR “complete resection” OR postoperative OR lobectomy) AND (“recurrence” OR relapse OR “pattern of recurrence” OR “metastatic pattern”) AND (“brain metastasis” OR “CNS metastasis” OR “intracranial metastasis” OR “brain recurrence”).

The full electronic search strategies for each database, including PubMed, Embase, and Web of Science, are provided in Appendices. Only English-language studies were included. Reference lists of eligible studies were manually screened to identify additional citations. However, we acknowledge that exclusion of non-English-language publications may introduce language bias and potentially limit the generalizability of the findings. Reference lists of eligible studies were manually screened to identify additional citations. Unpublished studies, conference abstracts, editorials, and non-peer-reviewed reports were excluded.

Eligibility criteria

Studies were evaluated against predefined inclusion and exclusion criteria focused on population, intervention, outcomes, and design characteristics.

Inclusion Criteria

We included studies enrolling adult patients (≥18 years) with histologically confirmed NSCLC who underwent curative-intent surgical resection (R0). Eligible reports were required to describe postoperative brain metastasis, either as the initial site of recurrence or as part of cumulative follow-up, and to examine associations between clinicopathological features and brain recurrence, or to provide information regarding incidence, management strategies, or survival outcomes. We considered observational designs including prospective and retrospective cohort studies, case-control analyses, registry-based studies, and post-hoc analyses of clinical trials.

Exclusion Criteria

Studies were excluded if they predominantly involved small-cell lung cancer or mixed histologies for which NSCLC-specific data were not extractable, or if surgery was non-curative (R1 or R2 resection). Additional exclusion criteria comprised studies focusing exclusively on synchronous brain metastasis or on non-brain recurrence, as well as animal studies, laboratory investigations, pediatric series, case reports with fewer than 10 patients, narrative reviews, editorials, and conference abstracts. When multiple publications derived from the same cohort were identified, the most complete or most recent analysis was selected.

Study selection

Study selection was performed in two stages. First, two independent reviewers screened titles and abstracts to assess potential eligibility. Subsequently, full texts of selected articles were examined in detail. Any discrepancies regarding study inclusion were resolved through consensus, and involvement of a third reviewer was arranged when necessary. The overall selection process followed the PRISMA recommendations.

Data extraction

Data extraction was carried out independently by two reviewers using a standardized data collection form. Extracted variables included study characteristics, patient demographics, pathological findings, perioperative treatments, incidence and timing of postoperative brain metastasis, and adjusted effect estimates-preferably hazard ratios (HRs) or odds ratios-from multivariable analyses. Only variables for which multivariable-adjusted estimates were reported were included in the pooled analyses of risk factors, to minimize confounding and ensure comparability across studies. Information on treatment modalities following recurrence and on survival outcomes was also collected, along with the duration of follow-up. When reported, molecular and radiological information, such as EGFR mutation, SUVmax, or specific imaging characteristics, was additionally extracted.

Data synthesis

When appropriate data were available, pooled effect estimates were synthesized using random-effects models. HRs or odds ratios (ORs) adjusted in multivariable analyses were prioritized. Statistical heterogeneity was assessed using the I² statistic and Cochran’s Q test. For proportion data of postoperative brain metastasis incidence, a Freeman-Tukey double arcsine variance-stabilizing transformation was applied prior to pooling. Incidence estimates were pooled as cumulative proportions and therefore did not explicitly account for between-study differences in follow-up duration, which varied across cohorts. Publication bias was assessed using funnel plots and Egger’s regression test when ≥10 studies were available. All meta-analyses were conducted using EZR (Easy R) (Yoshiharu Kanda, Jichi Medical University, Saitama, Japan), an updated graphical user interface for R (R Core Team, R Foundation for Statistical Computing, Vienna, Austria), which incorporates the meta (Guido Schwarzer, University of Freiburg, Freiburg, Germany) and metafor packages (Wolfgang Viechtbauer, Maastricht University, Maastricht, Netherlands).

Quality assessment

The methodological quality of included studies was assessed independently by two reviewers using the Newcastle-Ottawa Scale (NOS) for cohort studies, which evaluates selection, comparability, and outcome assessment (maximum score: nine points) [13]. Studies were considered to be of higher quality when they included well-defined surgical cohorts, adequate postoperative follow-up, radiologically confirmed brain metastasis, and multivariable adjustment for key clinicopathological factors. Discrepancies were resolved by consensus. Study quality was not used as an exclusion criterion but was considered when interpreting heterogeneity.

Results

Study Selection

A total of 727 records were initially identified through database searching, including 537 from Embase, 111 from Web of Science, and 79 from PubMed. After removal of 58 duplicates (54 identified automatically by Covidence and four through manual inspection), 669 records were screened based on titles and abstracts. Of these, 582 were excluded for not meeting eligibility criteria.

**Figure 1:**
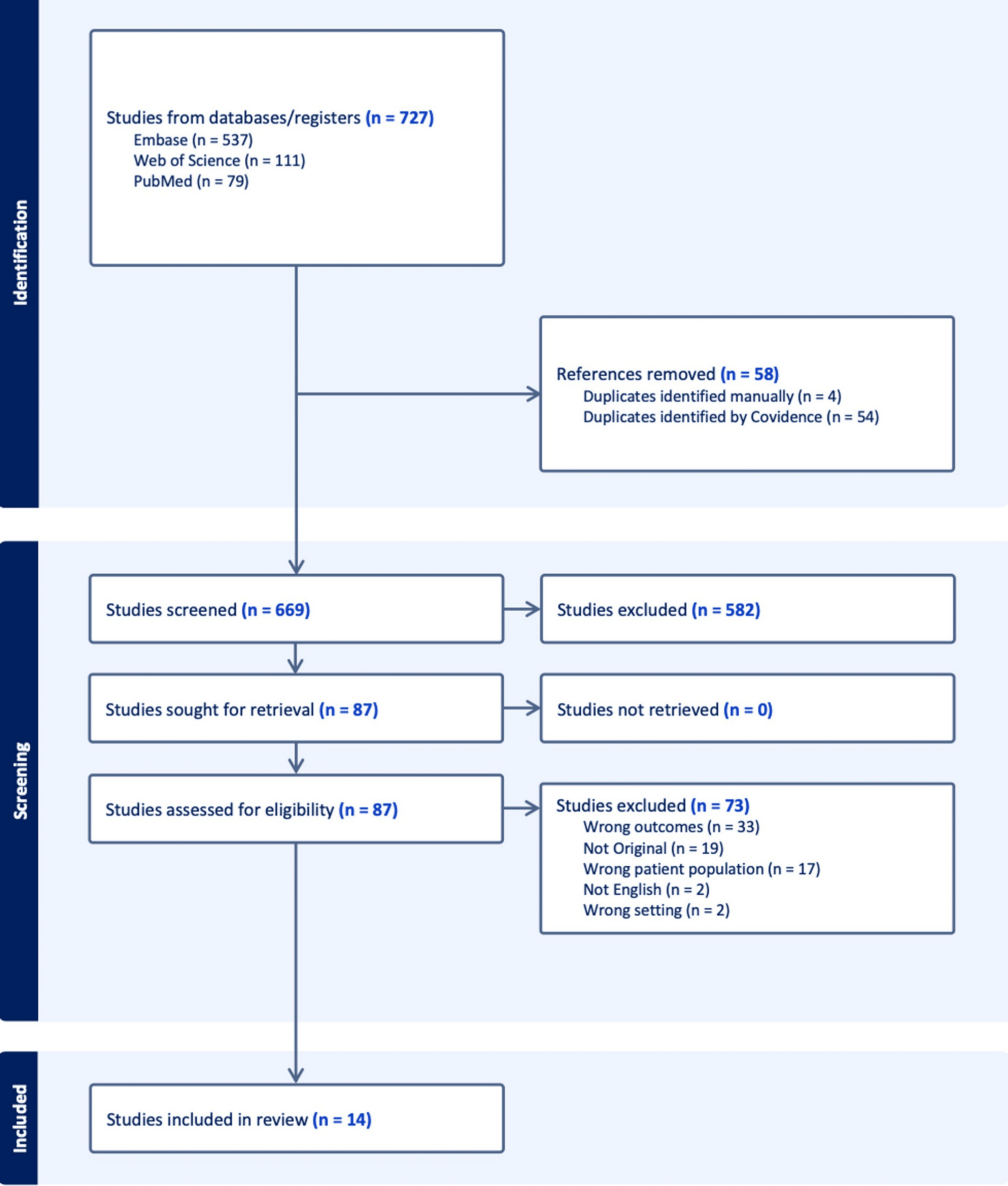
Selection flow

Eighty-seven full-text articles were assessed for eligibility. After a detailed review, 73 studies were excluded for the following reasons: unrelated outcomes (n = 33), non-original article types such as reviews or editorials (n = 19), ineligible patient population (n = 17), non-English language (n = 2), or inappropriate study setting (n = 2). Ultimately, 14 studies met the eligibility criteria and were included in the qualitative and quantitative synthesis. The study selection process is illustrated in the PRISMA flow diagram (Figure 1).

Characteristics of the Included Articles

A total of 14 retrospective cohort studies published between 2010 and 2025 were included, comprising 13,414 surgically resected NSCLC patients. Geographically, studies were predominantly conducted in Asia, including China (n = 9), Japan (n = 2), South Korea (n = 1), and Taiwan (n = 1), with only one study from the United States. All studies included patients undergoing curative-intent pulmonary resection, most commonly lobectomy with systematic lymph node dissection.

Study sample sizes ranged from 181 to 1,962, and postoperative metachronous brain metastasis events ranged from 30 to 135 per study, yielding a combined total of 883 intracranial recurrences, corresponding to an overall frequency of approximately 6.6% (883/13,414) during postoperative follow-up. Across included studies, brain metastasis was variably defined as either the first site of recurrence or as occurring at any time during follow-up; however, all analyses were restricted to metachronous (postoperative) intracranial events, and studies exclusively reporting synchronous brain metastasis were excluded. Given the limited number of studies consistently reporting first-site recurrence only, these definitions were pooled, and this heterogeneity is acknowledged as a potential source of between-study variability. Median follow-up duration ranged from 33 to 70 months in studies reporting this information.

Across the included articles, adenocarcinoma accounted for 45-100% of cases, and several studies exclusively evaluated adenocarcinoma cohorts. Only a minority of cohorts included substantial proportions of non-adenocarcinoma histology (approximately 20-55%). Several Asian studies reported high proportions of never-smoking patients (approximately 30-75%), consistent with the epidemiology of lung adenocarcinoma in East Asia.

Postoperative screening practices showed notable variation. Only four out of 14 studies (28.6%) incorporated routine scheduled brain MRI as part of follow-up, whereas the remaining studies relied exclusively on symptom-driven neuroimaging. This differential surveillance intensity represents a major source of detection bias. Cohorts without routine brain imaging are likely to under-detect subclinical intracranial metastases, which may lead to underestimation of postoperative brain metastasis incidence and attenuated or inconsistent associations in risk factor analyses. Consequently, variability in surveillance strategies likely contributed to heterogeneity in reported incidence estimates and may partially explain between-study differences in observed postoperative intracranial recurrence rates.

Molecular profiling was available in a subset of studies. Among publications reporting mutation status, EGFR positivity ranged from 55% to 85%, reflecting the high prevalence of driver alterations in Asian adenocarcinoma populations. Other biomarker information, such as SUVmax, was available only in limited cohorts (Table 1).

**Table 1:** Demographic data of the included articles All included studies exclusively enrolled adult patients with histologically confirmed NSCLC who underwent curative-intent surgical resection. Follow-up duration indicates the median or mean postoperative observation period when available. Brain metastasis events refer to metachronous intracranial recurrence detected during postoperative follow-up. Brain metastasis screening denotes whether routine brain imaging was implemented in the study protocol. Abbreviations: NR, not reported; NSCLC, non-small cell lung cancer

Author	Publication year	Country	Study design	Sample size	Brain metastasis event	Follow-up	Brain metastasis screening
Hubbs et al. [14]	2010	USA	Retrospective cohort	975	60	33 months	No
Kuo et al. [15]	2014	Taiwan	Retrospective cohort	181	61	NR	No
Won et al. [16]	2015	South Korea	Retrospective cohort	1218	87	43.6 months	No
Zhang et al. [17]	2016	China	Retrospective cohort	637	NR	38 months	Yes
Fu et al. [18]	2020	China	Retrospective cohort	272	35	NR	No
Li et al. [19]	2020	China	Retrospective cohort	332	135	70 months	Yes
Zhang et al. [20]	2020	China	Retrospective cohort	357	60	40.4 months	No
Liu et al. [21]	2021	China	Retrospective cohort	1962	30	NR	No
Deng et al. [22]	2021	China	Retrospective cohort	1512	118	53.2 months	Yes
Mizuno et al. [23]	2021	Japan	Retrospective cohort	1099	72	60.5 months	No
Li et al. [24]	2022	China	Retrospective cohort	181	64	≥60 months	Yes
Liu et al. [25]	2022	China	Retrospective cohort	888	62	54.6 months	No
Zhang et al. [26]	2025	China	Retrospective cohort	669	69	NR	No
Motono et al. [27]	2025	Japan	Retrospective cohort	1000	30	52.6 months	NR

Results of Meta-Analysis

**Figure 2:**
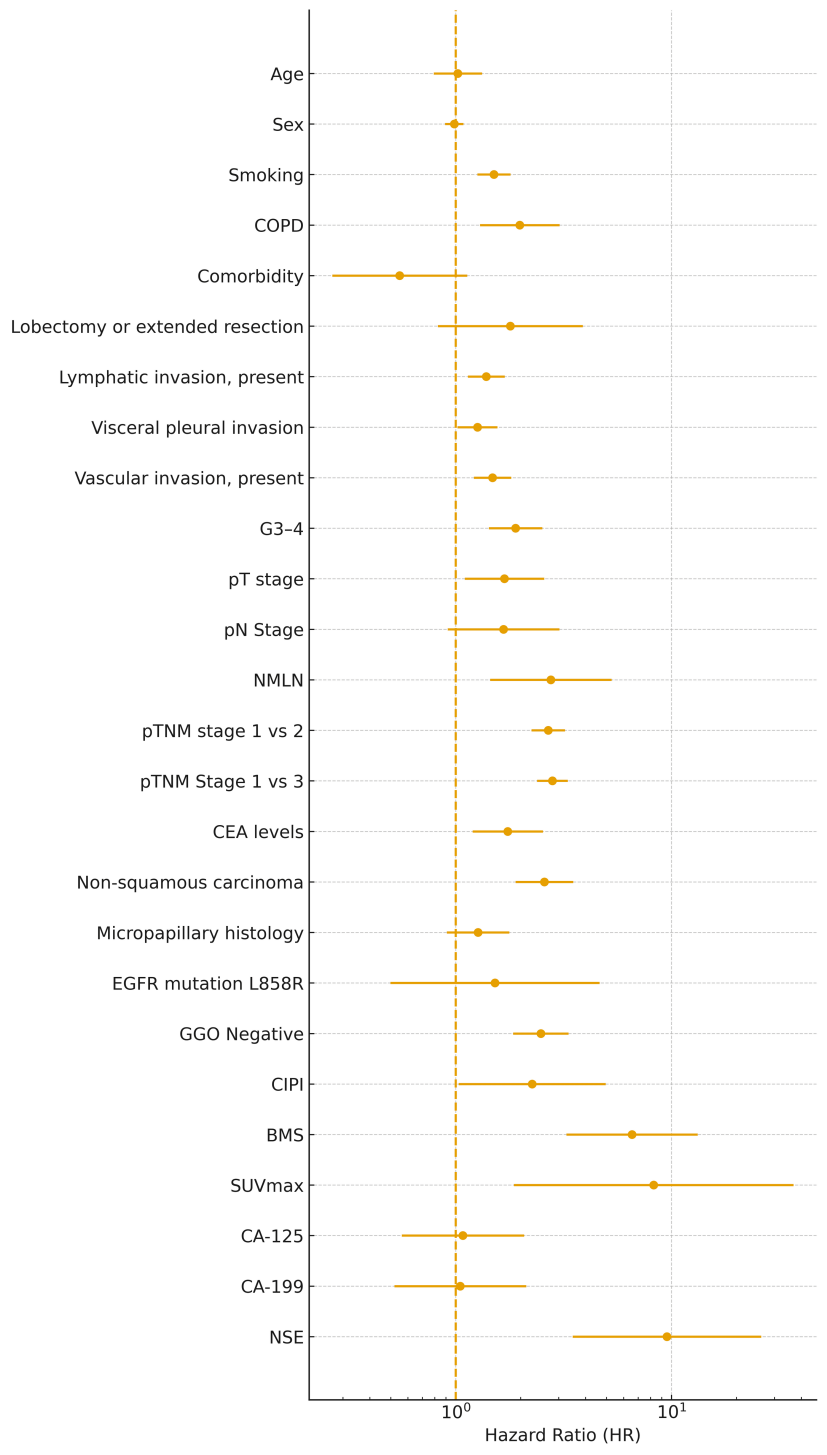
Forest plot illustrating pooled hazard ratios for risk factors associated with postoperative brain metastasis in NSCLC Forest plot showing pooled hazard ratios (HRs) with corresponding 95% confidence intervals (CIs) for each investigated risk factor in postoperative non-small cell lung cancer (NSCLC). Estimates were calculated using a random-effects model. A logarithmic scale was applied to the x-axis. The vertical dashed line represents HR = 1.0. Variables are ordered according to the predefined dataset order and displayed in reverse order from bottom to top for presentation purposes. Abbreviations: BMS, brain metastasis score; CA-125, cancer antigen-125; CA-199, carbohydrate antigen-19-9; CEA, carcinoembryonic antigen; CIPI, clinical and imaging prognostic index; COPD, chronic obstructive pulmonary disease; EGFR, epidermal growth factor receptor; G3-4, pathological grade 3-4; GGO, ground-glass opacity; NMLN, number of metastatic lymph nodes; NSE, neuron-specific enolase; pN, pathological N stage; pT, pathological T stage; pTNM, pathological TNM stage; SUVmax, maximum standardized uptake value; TNM, tumor-node-metastasis

A total of 14 studies investigating postoperative metachronous brain metastasis among patients with resected NSCLC were included in the meta-analysis. The pooled results demonstrated that several clinicopathological features were significantly associated with an increased risk of postoperative brain metastasis, whereas conventional demographic characteristics showed no consistent association. Forest plots for each risk factor are presented in Figure 2.

Pooled HRs for risk factors of postoperative brain metastasis in NSCLC are summarized in Table 2.

**Table 2:** Pooled hazard ratios for risk factors of postoperative brain metastasis in non-small cell lung cancer This table summarizes the random-effects meta-analysis of clinical, pathological, and treatment-related factors associated with postoperative brain metastasis in patients with non-small cell lung cancer (NSCLC). For each factor, pooled hazard ratios (HRs) and 95% confidence intervals (CIs) were calculated. Between-study heterogeneity was assessed using the I² statistic. Higher HRs indicate elevated risk of postoperative brain metastasis. Abbreviations: BMS, brain metastasis score; CA-125, cancer antigen-125; CA-199, carbohydrate antigen-19-9; CEA, carcinoembryonic antigen; CIPI, clinical and imaging prognostic index; COPD, chronic obstructive pulmonary disease; EGFR, epidermal growth factor receptor; G3-4, pathological grade 3-4; GGO, ground-glass opacity; NMLN, number of metastatic lymph nodes; NSE, neuron-specific enolase; pN, pathological N stage; pT, pathological T stage; pTNM, pathological TNM stage; SUVmax, maximum standardized uptake value; TNM, tumor-node-metastasis

Risk factor	Pooled HR	95% CI lower	95% CI upper	I² (%)
Age [14,25,27]	1.02	0.79	1.32	18.62
Sex [17,22-24]	0.98	0.89	1.09	18.78
Smoking [16,22]	1.50	1.26	1.79	0
COPD [15]	1.98	1.29	3.03	0
Comorbidity [21]	0.55	0.27	1.13	0
Lobectomy or extended resection [22,27]	1.79	0.83	3.88	0
Lymphatic invasion, present [14,21-23,27]	1.38	1.14	1.69	0
Visceral pleural invasion [22,23]	1.26	1.02	1.56	9.11
Vascular invasion, present [22,23,27]	1.48	1.21	1.81	0
G3-4 [17,21,23,25,27]	1.89	1.42	2.52	8.78
pT stage [14-17,21]	1.68	1.10	2.57	0
pN Stage [14,16,17,19,23,25,27]	1.66	0.92	3.02	67.48
NMLN [20,26]	2.76	1.44	5.28	58.24
pTNM stage 1 vs. 2 [17-19,22,25,26]	2.68	2.24	3.21	0
pTNM Stage 1 vs. 3 [17-19,22,25,26]	2.80	2.38	3.30	2.02
CEA levels [23,24]	1.74	1.20	2.54	0
Non-squamous carcinoma [15-17,20,24,26]	2.58	1.89	3.50	0
Micropapillary histology [19,22]	1.27	0.92	1.77	51.15
EGFR mutation L858R [25,26]	1.52	0.50	4.64	86.12
GGO Negative [22]	2.48	1.85	3.33	0
CIPI [27]	2.26	1.03	4.95	0
BMS [18]	6.56	3.26	13.21	0
SUVmax [27]	8.27	1.86	36.77	0
CA-125 [17]	1.08	0.56	2.08	0
CA-199 [17]	1.05	0.52	2.12	0
NSE [17]	9.52	3.48	26.04	0

Demographic and Baseline Clinical Characteristics

Age (analyzed as a continuous variable or binomial) was not significantly associated with metachronous brain metastasis (pooled HR = 1.02, 95% CI 0.79-1.32, I² = 18.6%). Similarly, male sex showed no significant association (HR = 0.98, 95% CI 0.89-1.09, I² = 18.8%). Smoking status (current/former vs never) was significantly associated with an increased risk of brain metastasis (HR = 1.50, 95% CI 1.26-1.79, I² = 0.0%). In contrast, chronic obstructive pulmonary disease (COPD) was evaluated in only one study, which reported a higher risk (HR = 1.98, 95% CI 1.29-3.03). Comorbidity burden (comorbidity score) was also reported by a single study and suggested a non-significant tendency towards reduced risk (HR = 0.55, 95% CI 0.27-1.13), although precision was limited.

Tumor Histology and Pathological Differentiation

Non-squamous carcinoma histology was strongly associated with a higher incidence of brain metastasis (HR = 2.58, 95% CI 1.89-3.50, I² = 0.0%). Within this group, micropapillary histology showed a tendency towards increased risk but did not reach statistical significance (HR = 1.27, 95% CI 0.91-1.77, I² = 51.1%). Poorly differentiated tumors (G3-4) were significantly associated with brain metastasis (HR = 1.89, 95% CI 1.42-2.52, I² = 8.8%), indicating that histological grade is an important determinant.

Invasion-Related Pathological Features

Lymphatic invasion was one of the most consistent pathological predictors. The presence of lymphatic invasion significantly increased the risk of brain metastasis (HR = 1.39, 95% CI 1.14-1.69, I² = 0.0%). Visceral pleural invasion was also significantly associated with brain metastasis (HR = 1.26, 95% CI 1.02-1.56, I² = 9.1%). Similarly, vascular invasion conferred a significantly elevated risk (HR = 1.48, 95% CI 1.21-1.81, I² = 0.0%). These findings indicate that invasion beyond the primary tumor, particularly into lymphatic and vascular structures and the visceral pleura, is consistently linked to postoperative brain relapse.

Tumor Size, T Category, and Lymph Node Involvement

Pathological T category was significantly associated with metachronous brain metastasis (HR = 1.68, 95% CI 1.10-2.57, I² = 0.0%), suggesting that more locally advanced primary tumors are at higher risk. Pathological N stage showed a trend toward increased risk, but the pooled estimate was not statistically significant and exhibited substantial heterogeneity (HR = 1.66, 95% CI 0.92-3.02, I² = 67.5%). This heterogeneity may reflect differences across studies in nodal staging systems, extent of lymph node dissection, pathological assessment, and use of adjuvant therapies, all of which could influence both nodal classification and subsequent risk of intracranial recurrence. In contrast, a more quantitative assessment of nodal burden demonstrated a clearer association: the number of metastatic lymph nodes (NMLN) was significantly associated with brain metastasis (HR = 2.76, 95% CI 1.44-5.28, I² = 58.2%). Lymph node-related composite indices such as CIPI and BMS were each evaluated in single studies and showed elevated risks (CIPI: HR = 2.26, 95% CI 1.03-4.95; BMS: HR = 6.56, 95% CI 3.26-13.21).

Pathological TNM Stage

A higher pathological TNM stage was consistently associated with a substantially increased risk of brain metastasis. Compared with stage I, pathological stage II had a pooled HR of 2.68 (95% CI 2.24-3.21, I² = 0.0%), and stage III had a pooled HR of 2.80 (95% CI 2.38-3.30, I² = 2.0%). These findings highlight that overall pathological stage, integrating T and N status, is a robust stratifier of brain metastasis risk after surgery.

Surgical Procedure

The extent of resection was evaluated in terms of lobectomy or extended resection. Patients undergoing lobectomy or extended resection showed a non-significant trend towards higher risk (HR = 1.79, 95% CI 0.83-3.88, I² = 0.0%). Segmentectomy and wedge resection were reported less consistently and could not be pooled.

Molecular Marker: EGFR Mutation

EGFR mutation (L858R) was evaluable in two studies. Other EGFR mutation subtypes, including exon 19 deletion, were not pooled because they were either inconsistently reported or lacked multivariable-adjusted effect estimates across studies. The pooled analysis for L858R yielded an HR of 1.52 (95% CI 0.50-4.64, I² = 86.1%). Although the point estimate suggested a higher risk of metachronous brain metastasis among patients with EGFR-mutated tumors, the wide confidence interval and substantial heterogeneity preclude definitive conclusions. These results nonetheless indicate that EGFR mutation may contribute to brain tropism, warranting further investigation in larger, standardized cohorts.

Serum Biomarkers and Metabolic Parameters

Among serum biomarkers, elevated CEA levels were significantly associated with increased risk of brain metastasis (HR = 1.74, 95% CI 1.20-2.54, I² = 0.0%). Other tumor markers were each reported in single studies: CA-125 (HR = 1.08, 95% CI 0.56-2.08), CA19-9 (HR = 1.05, 95% CI 0.52-2.12), and NSE (HR = 9.52, 95% CI 3.48-26.04). While NSE showed markedly elevated HR, the evidence is currently limited to a single study. Maximum standardized uptake value (SUVmax) on PET was also examined in a single study. It showed a considerable increase in risk (HR = 8.27, 95% CI 1.86-36.77), suggesting that high metabolic activity might be linked with subsequent brain metastasis.

Imaging-Related and Composite Risk Indices

Ground-glass opacity (GGO) status was reported in one study, where the absence of GGO (GGO negative) was associated with a higher risk of brain metastasis (HR = 2.48, 95% CI 1.85-3.33). Two composite indices, the clinical prognostic index (CIPI) and brain metastasis score (BMS), also showed significant associations in single studies (CIPI HR = 2.26, 95% CI 1.03-4.95; BMS HR = 6.56, 95% CI 3.26-13.21), indicating the potential utility of multivariable risk stratification tools, although external validation remains limited.

Quality Assessment 

Quality assessment was conducted using the NOS for observational cohort studies, focusing on three domains: selection, comparability, and outcome assessment. All 14 studies adopted a retrospective cohort design and met the criteria for adequate cohort selection, including histologically confirmed NSCLC and curative-intent resection. Most studies reported well-defined eligibility criteria and postoperative follow-up periods sufficient for detecting metachronous brain metastasis (median follow-up 33-70 months).

Comparability between exposure groups was generally acceptable, as the majority of studies adjusted for key prognostic variables such as pathological stage, tumor differentiation, lymphovascular invasion, and smoking status in multivariable analyses. However, the extent of covariate adjustment varied among studies, and only a subset incorporated molecular markers or postoperative therapy in their multivariable models.

Outcome assessment was based primarily on radiologic confirmation of postoperative brain metastasis across studies, although brain imaging strategies differed substantially. Several studies relied on symptom-triggered neuroimaging, whereas others incorporated routine postoperative brain MRI, potentially introducing detection bias. The definition of postoperative brain failure (initial versus cumulative site of recurrence) also varied, contributing to heterogeneity among reported outcomes (Table 3).

**Table 3:** Quality assessment of the included studies using the Newcastle-Ottawa Scale (NOS) Each study was evaluated on three methodological domains: selection (maximum four points), comparability (maximum two points), and outcome assessment (maximum three points), giving a total possible score of nine. Higher scores indicate greater methodological quality. All included studies were conducted using retrospective cohort designs and focused on patients who underwent curative-intent surgical resection for non-small cell lung cancer.

Author	Year	Selection	Comparability	Outcome	Total NOS score
Hubbs et al. [14]	2010	3	1	2	6
Kuo et al. [15]	2014	3	1	2	6
Won et al. [16]	2015	3	1	2	6
Zhang et al. [17]	2016	3	1	2	6
Fu et al. [18]	2020	3	1	2	6
Li et al. [19]	2020	4	2	2	8
Zhang et al. [20]	2020	3	1	2	6
Liu et al. [21]	2021	4	2	2	8
Deng et al. [22]	2021	4	2	2	8
Mizuno et al. [23]	2021	4	2	2	8
Li et al. [24]	2022	4	2	2	8
Liu et al. [25]	2022	4	2	2	8
Zhang et al. [26]	2025	4	2	2	8
Motono et al. [27]	2025	4	2	2	8

Discussion

Summary of the Study

In this systematic review and meta-analysis involving 14 retrospective cohort studies and more than 13,000 patients undergoing curative-intent resection for NSCLC, we examined the incidence and determinants of postoperative metachronous brain metastasis. Across included studies, adenocarcinoma was the dominant histological subtype, and the majority of patients were treated in Asian surgical cohorts with long-term follow-up. Although the reported incidence of postoperative brain metastasis varied owing to differences in imaging strategies and study design, our findings confirm that metachronous brain recurrence remains a clinically relevant pattern of disease progression, particularly among patients with adverse clinicopathological features. Furthermore, several molecular characteristics-including EGFR mutation-have been investigated as potential predictors of intracranial failure; however, the available evidence remains inconsistent and highly heterogeneous, and pooled estimates did not demonstrate a robust or reproducible association. In contrast, clinicopathological factors showed more consistent and stable associations across multivariable analyses, suggesting that molecular predictors alone are currently insufficient for reliable postoperative risk stratification of brain recurrence.

Given that a proportion of postoperative brain metastases are detected only after neurological presentation, our findings highlight the potential clinical relevance of risk-adapted postoperative neuroimaging. However, this concept should be regarded as hypothesis-generating, as none of the included studies directly evaluated surveillance strategies or compared scheduled versus symptom-driven brain imaging. In particular, whether scheduled brain imaging may benefit selected high-risk individuals remains an important unanswered question and represents a key motivation for further prospective investigation.

Comparison With Other Studies

Prior systematic reviews have described recurrence patterns following resection for early-stage NSCLC, yet few have focused specifically on metachronous brain metastasis as the primary outcome [28-30]. Several studies have reported higher rates of brain recurrence in adenocarcinoma and EGFR-mutant populations, reflecting the changing epidemiology of NSCLC in East Asian cohorts [31,32]. Our findings are partially consistent with these observations; however, the magnitude and consistency of association between EGFR mutation and postoperative brain metastasis were less pronounced in our pooled analysis compared with earlier reports. These discrepancies may be attributable to differences in study populations, evolving molecular testing strategies, and competing pathological determinants that were simultaneously evaluated in multivariable models.

Conversely, several clinicopathological and host-related factors that have received comparatively less attention in prior recurrence analyses demonstrated more robust and consistent associations in our synthesis. Specifically, lymph node involvement, visceral pleural invasion, tumor differentiation, smoking status, and the presence of chronic pulmonary comorbidity, such as COPD, emerged as independent predictors of metachronous intracranial failure across multiple adjusted models [33-35]. These observations suggest that classical pathological and clinical factors continue to play a central role in determining brain recurrence risk even in the molecular era, and that reliance solely on genomic markers may underestimate the contribution of fundamental disease biology and host susceptibility.

Notably, the absence of routine postoperative brain imaging in earlier cohorts likely led to an underestimation of intracranial relapse, underscoring the need for risk-adapted surveillance strategies [36]. Future studies should therefore integrate standardized imaging protocols, contemporary biomolecular testing, and comprehensive adjustment for clinicopathological determinants to clarify the relative prognostic weight of genomic versus traditional risk factors in surgically treated NSCLC.

Strengths of the Study

This review synthesizes contemporary evidence across 15 years of surgical oncology practice, integrating clinical, pathological, radiologic, and molecular determinants of postoperative brain failure. The inclusion of more than 13,000 resected NSCLC cases enables risk estimation in real-world surgical cohorts across different regions and healthcare systems. Rigorous study selection, predefined eligibility criteria, and structured quality assessment provide a reliable methodological foundation for interpreting pooled estimates.

Importantly, by focusing exclusively on metachronous brain metastasis following curative-intent surgery, our analysis addresses a clinically relevant gap that has been insufficiently characterized in previous recurrence studies. Although EGFR mutation has long been recognized as a potential driver of brain tropism in NSCLC, our findings suggest that the association may be less consistent than generally assumed in the surgical setting, particularly when compared with fundamental pathological and clinical features [37]. Lymph node involvement, visceral pleural invasion, histologic differentiation, smoking history, and comorbid conditions such as COPD demonstrated more consistent associations with postoperative brain recurrence across multivariable analyses. These results highlight that traditional tumor biology and host-related clinical factors remain key determinants of intracranial relapse risk beyond molecular profile alone, underscoring the need for future models that integrate both classical clinicopathological parameters and contemporary genomic information. Such integrated risk stratification has important clinical implications for multidisciplinary postoperative planning, including coordination among thoracic surgeons, medical oncologists, radiation oncologists, and radiologists when considering follow-up strategies and early intervention for high-risk patients.

Limitations

Several limitations inherent to the available literature should be recognized. First, all included studies were retrospective observational cohorts, which may introduce selection bias and residual confounding despite multivariable adjustment in most analyses. Second, considerable heterogeneity existed in postoperative brain imaging strategies. While a minority of studies incorporated scheduled brain MRI surveillance, the majority relied on symptom-driven neuroimaging, which may have led to delayed detection of subclinical intracranial recurrence and an underestimation of the true metachronous incidence. The variability in imaging modality, surveillance interval, and clinical threshold for neuroimaging likely contributed to detection bias and heterogeneity across studies. In addition, differences in follow-up duration and timing of recurrence assessment may have introduced time-to-event bias, and competing risks, particularly postoperative mortality, were not uniformly accounted for across studies. These factors may have influenced both incidence estimates and HR calculations in postoperative cohorts.

Third, although effect estimates were derived primarily from multivariable analyses, the selection of covariates included in these models differed substantially among studies. Some models adjusted for classical pathological parameters such as nodal stage, tumor differentiation, and lymphovascular invasion. In contrast, others incorporated molecular markers or adjuvant treatment variables, resulting in inconsistent adjusted HRs. As a result, pooled estimates reflect partially harmonized effect sizes rather than fully standardized prognostic comparisons and should therefore be interpreted with caution.

Fourth, molecular and genomic profiling was inconsistently reported, and even among studies with available genetic data, the inclusion of biomarkers (such as EGFR, PD-L1, or other driver alterations) in multivariable models varied markedly. Likewise, reporting of adjuvant chemotherapy and radiotherapy was heterogeneous, precluding comprehensive evaluation of treatment-related modifiers of brain recurrence.

Finally, most included cohorts originated from East Asian populations, in which the prevalence of adenocarcinoma and EGFR-mutant disease is significantly higher than in Western populations. These epidemiologic and biological differences may limit the generalizability of our findings and highlight the need for future prospective studies including diverse populations and standardized neuro-oncological follow-up protocols. In particular, external validation in Western and multi-ethnic cohorts will be essential to confirm the applicability of these risk factors across different healthcare systems and genomic backgrounds [38].

## Conclusions

Metachronous brain metastasis represents a clinically relevant pattern of recurrence after curative-intent resection for NSCLC. Robust clinicopathological factors-such as nodal involvement, visceral pleural invasion, tumor differentiation, and adenocarcinoma histology-were most consistently associated with postoperative intracranial relapse in multivariable analyses. In contrast, molecular characteristics, including EGFR mutation, showed heterogeneous and inconclusive associations, limiting their current utility as standalone predictors. These findings support the development of personalized postoperative surveillance strategies primarily grounded in clinicopathological risk stratification. In this context, whether selected high-risk patients may benefit from scheduled, rather than purely symptom-driven, postoperative brain imaging remains an important hypothesis-generating clinical question. Prospective, multi-center studies incorporating uniform imaging protocols and comprehensive molecular evaluation will be required to refine risk stratification and to determine whether intensified neurological follow-up can improve early detection and outcomes in resected NSCLC.

## Appendices

PubMed

(“lung cancer” OR “non small cell lung cancer” OR NSCLC) AND (“resection” OR “surgical resection” OR “complete resection” OR “postoperative” OR “lobectomy”) AND (“recurrence” OR relapse OR “pattern of recurrence” OR “metastatic pattern”) AND (“brain metastasis” OR “CNS metastasis” OR “intracranial metastasis” OR “brain recurrence”)

Filters applied: English language; human studies.

Embase

(“lung cancer” OR “non small cell lung cancer” OR NSCLC) AND (“resection” OR “surgical resection” OR “complete resection” OR “postoperative” OR “lobectomy”) AND (“recurrence” OR “relapse” OR “pattern of recurrence” OR “metastatic pattern”) AND (“brain metastasis” OR “CNS metastasis” OR “intracranial metastasis” OR “brain recurrence”)

Filters applied: English language; human studies.

Web of Science

TS=(“lung cancer” OR “non small cell lung cancer” OR NSCLC) AND TS=(“resection” OR “surgical resection” OR “complete resection” OR “postoperative” OR “lobectomy”) AND TS=(“recurrence” OR relapse OR “pattern of recurrence” OR “metastatic pattern”) AND TS=(“brain metastasis” OR “CNS metastasis” OR “intracranial metastasis” OR “brain recurrence”)

Filters applied: English language.

## References

[1] Araghi M, Mannani R, Heidarnejad Maleki A (2023). Recent advances in non-small cell lung cancer targeted therapy; an update review. Cancer Cell Int.

[2] Schabath MB, Cote ML (2019). Cancer progress and priorities: lung cancer. Cancer Epidemiol Biomarkers Prev.

[3] Bhamani A, Creamer A, Verghese P (2025). Low-dose CT for lung cancer screening in a high-risk population (SUMMIT): a prospective, longitudinal cohort study. Lancet Oncol.

[4] Philip B, Jain A, Ramesh P (2023). Follow up and surveillance post lung cancer surgery: a narrative review. Video-Assist Thorac Surg.

[5] Yri OE, Astrup GL, Karlsson AT (2025). Survival and quality of life after first-time diagnosis of brain metastases: a multicenter, prospective, observational study. Lancet Reg Health Eur.

[6] Wang S, Tang W, Jin F, Luo H, Yang H, Wang Y (2025). Comprehensive analysis of lung cancer metastasis: sites, rates, survival, and risk factors—a systematic review and meta-analysis. Clin Respir J.

[7] Huang Q, Li Y, Huang Y (2025). Advances in molecular pathology and therapy of non-small cell lung cancer. Signal Transduct Target Ther.

[8] Shimizu R, Kinoshita T, Sasaki N (2020). Clinicopathological factors related to recurrence patterns of resected non-small cell lung cancer. J Clin Med.

[9] Wang C, Wu Y, Shao J, Liu D, Li W (2020). Clinicopathological variables influencing overall survival, recurrence and post-recurrence survival in resected stage I non-small-cell lung cancer. BMC Cancer.

[10] Franchino F, Rudà R, Soffietti R (2018). Mechanisms and therapy for cancer metastasis to the brain. Front Oncol.

[11] Gondi V, Bauman G, Bradfield L (2022). Radiation therapy for brain metastases: an ASTRO clinical practice guideline. Pract Radiat Oncol.

[12] Page MJ, McKenzie JE, Bossuyt PM (2021). The PRISMA 2020 statement: an updated guideline for reporting systematic reviews. BMJ.

[13] Stang A (2010). Critical evaluation of the Newcastle-Ottawa scale for the assessment of the quality of nonrandomized studies in meta-analyses. Eur J Epidemiol.

[14] Hubbs JL, Boyd JA, Hollis D, Chino JP, Saynak M, Kelsey CR (2010). Factors associated with the development of brain metastases: analysis of 975 patients with early stage nonsmall cell lung cancer. Cancer.

[15] Kuo CH, Wu CY, Lee KY (2014). Chronic obstructive pulmonary disease in stage I non-small cell lung cancer that underwent anatomic resection: the role of a recurrence promoter. COPD.

[16] Won YW, Joo J, Yun T (2015). A nomogram to predict brain metastasis as the first relapse in curatively resected non-small cell lung cancer patients. Lung Cancer.

[17] Zhang F, Zheng W, Ying L, Wu J, Wu S, Ma S, Su D (2016). A nomogram to predict brain metastases of resected non-small cell lung cancer patients. Ann Surg Oncol.

[18] Fu F, Zhang Y, Gao Z (2020). Development and validation of a five-gene model to predict postoperative brain metastasis in operable lung adenocarcinoma. Int J Cancer.

[19] Li C, Shen Y, Hu F (2020). Micropapillary pattern is associated with the development of brain metastases and the reduction of survival time in EGFR-mutation lung adenocarcinoma patients with surgery. Lung Cancer.

[20] Zhang Q, Cai XW, Feng W, Yu W, Fu XL (2020). Risk factors of brain metastases as initial failure in completely resected stage IIIA(N2) non-small cell lung cancer. Ann Transl Med.

[21] Liu X, Sun K, Yang F, Sui X, Jiang G, Wang J, Li X (2021). Different pathologic types of early stage lung adenocarcinoma have different post-operative recurrence patterns. Thorac Cancer.

[22] Deng C, Zhang Y, Ma Z, Fu F, Deng L, Li Y, Chen H (2021). Prognostic value of epidermal growth factor receptor gene mutation in resected lung adenocarcinoma. J Thorac Cardiovasc Surg.

[23] Mizuno T, Konno H, Nagata T, Isaka M, Ohde Y (2021). Osteogenic and brain metastases after non-small cell lung cancer resection. Int J Clin Oncol.

[24] Li J (2022). Risk factor of brain metastases and its influence on patient prognosis after complete resection of non-small cell lung cancer. Am J Transl Res.

[25] Liu X, Li X, Zhang C (2022). EGFR mutation is not a prognostic factor for CNS metastasis in curatively resected lung adenocarcinoma patients. Lung Cancer.

[26] Zhang J, Luo Z, Xie Z (2025). Association between EGFR mutation types and incidence of brain metastases in postoperative patients with stage I-III NSCLC. Tumori.

[27] Motono N, Mizoguchi T, Ishikawa M, Iwai S, Iijima Y, Uramoto H (2025). Prognostic impact of recurrence pattern for surgically resected non-small cell lung cancer. Oncology.

[28] Jiang C, Zhang Y, Deng P, Lin H, Fu F, Deng C, Chen H (2024). The overlooked cornerstone in precise medicine: personalized postoperative surveillance plan for NSCLC. JTO Clin Res Rep.

[29] Kudo Y, Haymaker C, Zhang J (2019). Suppressed immune microenvironment and repertoire in brain metastases from patients with resected non-small-cell lung cancer. Ann Oncol.

[30] Sonoda D, Kondo Y, Tamagawa S (2025). Characteristics of oligo-recurrence and treatment selection in non-small cell lung cancer. Cancers (Basel).

[31] Midha A, Dearden S, McCormack R (2015). EGFR mutation incidence in non-small-cell lung cancer of adenocarcinoma histology: a systematic review and global map by ethnicity (mutMapII). Am J Cancer Res.

[32] Burr R, Leshchiner I, Costantino CL (2024). Developmental mosaicism underlying EGFR-mutant lung cancer presenting with multiple primary tumors. Nat Cancer.

[33] Ohta R, Ryu Y, Tanaka K, Sano C, Hayashi H (2025). Peritoneal metastasis in non-small cell lung cancer: a systematic review of clinical features, molecular profiles, diagnostic approaches, and outcomes. Cancer Manag Res.

[34] Minamoto F, Araújo P, D'Ambrosio P, Dela Vega A, Lauricella L, Pêgo-Fernandes P, Terra R (2024). The association of visceral pleural invasion with skip N2 metastasis on clinical stage IA NSCLC. Clinics (Sao Paulo).

[35] Takada I, Shimada Y, Mimae T (2026). Prognosis, imaging characteristics, and clinicopathological features of heavy smokers with clinical stage I lung adenocarcinoma: a multicenter study. Gen Thorac Cardiovasc Surg.

[36] Mayer N, Boschetti L, Scarci M (2025). Brain imaging in patients with non-small cell lung cancer—a systematic review. J Clin Med.

[37] Jacome MA, Wu Q, Chen J, Mohamed ZS, Mokhtari S, Piña Y, Etame AB (2025). Molecular underpinnings of brain metastases. Int J Mol Sci.

[38] Ohta R, Sano C (2024). Disparity of the treatment of unresectable non-small cell lung cancer regarding chemotherapy: a systematic review and meta-analysis. Cureus.

